# Implementation and Evaluation of an Educational Program for Increasing Diversity and Inclusion in Surgery for Preclinical Students

**DOI:** 10.1001/jamanetworkopen.2020.15675

**Published:** 2020-09-01

**Authors:** Tyler S. Bryant, Anna L. Carroll, Jecca Rhea Steinberg, Paloma Marin-Nevarez, Tiffany N. Anderson, Sylvia Bereknyei Merrell, James N. Lau

**Affiliations:** 1 Stanford University School of Medicine, Stanford, California; 2 Stanford Surgery ACS Education Institute/Goodman Surgical Education Center, Department of Surgery, Stanford University, Stanford, California; 3 Stanford-Surgery Policy Improvement Research and Education Center, Department of Surgery, Stanford University, Palo Alto, California

## Abstract

This survey study assesses the student-led Service Through Surgery model for increasing diversity and inclusion in surgical education.

## Introduction

Increasing physician diversity is associated with improved care for underserved patients,^1^ but general surgery and its subspecialties remain less diverse than most specialties.^2^ Few interventions exist to address this issue during medical school. Programming that increases exposure to diverse, service-oriented mentors may inspire interest in surgical careers among underrepresented preclinical students.^3^ We describe the implementation and assessment of the student-led Service Through Surgery (STS) seminars,^4^ a replicable model for increasing diversity and inclusion in academic surgical education to guide other institutions wishing to incorporate similar interventions into their preclinical curriculum.

## Methods

For this survey study, from January 12, 2018, to March 15, 2019, medical student researchers interviewed surgeons from various specialties; departmental leadership positions; stages of training; and diverse racial/ethnic, sexual, and gender minority backgrounds to develop the STS course. Course objectives (Figure) included facilitating mentorship and career planning while emphasizing diversity, inclusion, and service. Presenters delivered lectures or interactive sessions facilitated by the student leaders. Presenters discussed health justice and advocacy topics, including how their backgrounds influenced their career path and practice. We recruited preclinical medical and physician assistant students for STS through emails and in-person announcements at a single institution. This study was approved by the Stanford University institutional review board. Verbal informed consent was obtained from all participants. This study followed the American Association for Public Opinion Research (AAPOR) reporting guideline.

**Figure.**
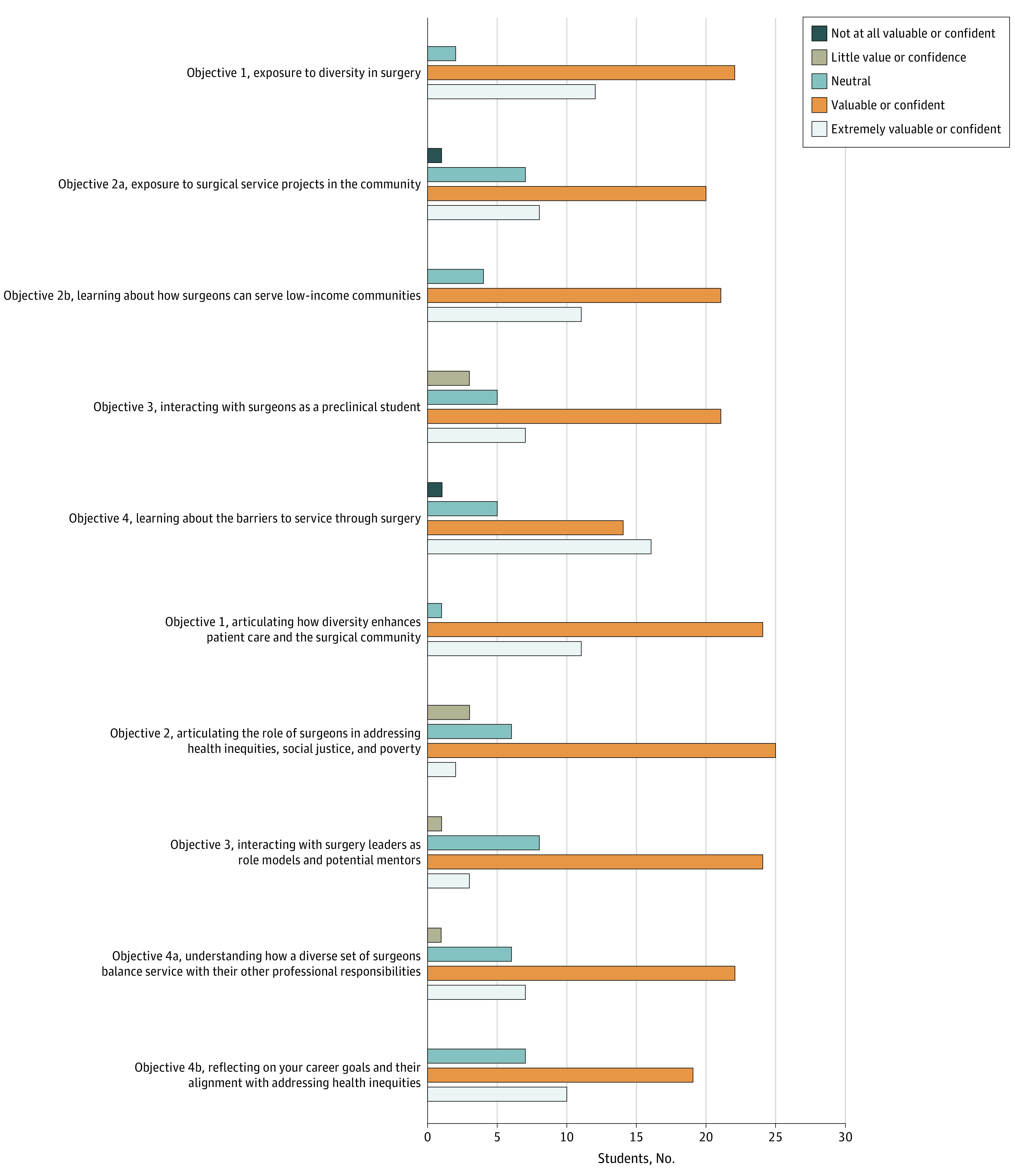
Student Responses to Postcourse Survey Questions Responses were scored on a 5-point Likert scale regarding the value of various attributes of the Service Through Surgery course and their confidence in meeting its learning objectives.

An anonymized postcourse survey (eFigure in the Supplement) assessed participants’ confidence in and value of course objectives on a 5-point Likert scale. Open-ended responses were analyzed through a systematic qualitative process.^5^ We developed a codebook based on course objectives and conducted deductive coding and theme analysis^5^ using Dedoose software, including assessing interrater reliability (Cohen κ, 0.75).^6^

## Results

The STS seminars took place in 2018 (8 sessions) and 2019 (9 sessions), with seminars led by 7 in-house attending surgeons, 2 general surgery residents, and 1 clinical medical student; 1 additional attending surgeon visited from a nearby institution. Presenters included 6 women (55%) and 8 individuals self-identifying as racial/ethnic minorities (73%). Of 47 student participants over the 2-year course duration (32 in 2018, 15 in 2019), 36 (77%) completed course evaluations: 22 (61%) were women, 23 (64%) self-identified as racial/ethnic minorities, and 12 (33%) were self-described disadvantaged students. Class years included 20 first year medical students (56%), 5 first year physician assistant students (14%), 11 second year medical students (30%).

Most participants were “confident” or “extremely confident” that the course met learning objectives (27 [75%]) and found the course “valuable” or “extremely valuable” (28 [78%]) (Figure). Regarding class format, 31 participants (86%) ranked lectures and discussions as “very valuable” and or 33 (92%) as “most valuable.” Two participants (6%) indicated that the course should be required, and 32 (89%) would either “somewhat strongly” or “strongly” recommend it to peers. Regarding mentorship, 12 students (33%) reported contacting or planning to contact a surgeon speaker for career advice or research collaboration.

Participants valued connecting with diverse surgeon speakers, often for the first time, based on important shared identities of language, cultural background, and sexual orientation. Participants associated newfound appreciation for their underrepresented background in surgery and an improved understanding of how to balance a demanding surgical career with providing care for underserved patients or conducting health disparities research (Box).

Box. **Exemplary Quotes Representing Themes From Qualitative Analysis of Student Reflections on Service Through Surgery Course Learning Objectives in Open-ended Survey Responses**

**Diversity in Surgery**

- Seeing value in diverse identities and backgrounds
  - “This course showed me how surgeons can leverage their background, identities, and passion to be of service to particular groups they identify with or feel passionately about.”
  - “[A pediatric surgeon in a Spanish language clinic’s] talk about culture and language-appropriate care tells me that *my upbringing can be an advantage in my future surgical care*...it was an ‘aha’ moment that showed me the importance of sharing my culture, language, and self in general with my patients.”

**Service Toward Underserved Populations**

- Broadened definition of service
  - “This course highlighted the fact that...surgeons [can improve] community health...not only by traveling abroad and empowering/training surgeons in resource-poor countries, but also by becoming more involved in their local communities...or in research to better understand the scope, drivers, and potential solutions for inequities in surgical care across race and socioeconomic status.”
  - “It definitely showed me different ways surgeons can be involved [in service], ranging from patient selection (in a few different ways) to research, to policies. Seeing the variety was important.”
  - “...that *service is not separate from work but integral to your practice*...represents a powerful idea that I will carry forward.”
- Ability to balance service with other responsibilities
  - *“*Previous[ly], I could not have named specific ways...surgeons could be involved in the community. Now I...have specific examples [and] understand that *service and surgery are not mutually exclusive*.”
  - “...highly successful, prominent surgeons can incorporate [service] into their work without drastic sacrifices made on their careers.”

**Mentorship Opportunities**

- Importance of shared identity
  - “As a female, [a]...benefit from this class was the opportunity to learn from senior female surgeons on how they navigate life.”
  - “*Seeing someone achieve so much career and service-wise* while maintaining a rich family life and *being very open about their queer identity was so inspiring*.”
- Aspects of a valuable mentor
  - “[I appreciated] when [a female thoracic surgeon] talked about how mentorship in medical school is different [in that] ‘you need a mentor who will advocate for you.’”

**Career Planning and Barriers to Service**

- Acknowledgment of obstacles and challenges
  - “...now I see that, while it can be difficult to find the time and support to implement service projects, *there are a lot of ways to integrate your passions into your work*.”
  - “The course...showed me how it can be difficult to help a population if you are not willing to devote a massive amount of personal effort (such as moving your family).”
- Increased interest in pursuing a surgical career
  - “It made me feel more comfortable going into surgery knowing that there are ways to provide for and support underserved communities.”
  - “*It made me more confident that I could become a surgeon* and still focus on and have an impact in helping work to decrease health disparities.”

## Discussion

Although many students aspire to help people by pursuing a career in health care, there is a dearth of tools that enable students to envision how to make meaningful contributions to the lives of underserved patients. The presenters of STS mentored and empowered potential future surgeons. Course objectives were met, and students found the course valuable. Although participant self-selection is a potential limitation of this study, many students broadened their definition of surgery to encompass a variety of service opportunities and to incorporate their own background and values. The speakers’ diversity and commitment to addressing health inequities inspired students to identify with a career in surgery. The STS program represents a replicable model that medical schools can adapt in pursuit of increasing diversity, inclusion, and service in the surgical workforce.

## Notes

Supplement. **eFigure.** Course development timeline for “Service Through Surgery” including questions asked to surgeons, discussion topics presented in the course, and a sample of the end-of-course evaluation survey
