# Supplementary material for: Implementation and Evaluation of an Educational Program for Increasing Diversity and Inclusion in Surgery for Preclinical Students
Source: JAMA Netw Open. 2020 Sep 1;3(9):e2015675. doi: 10.1001/jamanetworkopen.2020.15675 (PMC7489849; doi:10.1001/jamanetworkopen.2020.15675)
Supplement: Supplement. — eFigure. Course development timeline for “Service Through Surgery” including questions asked to surgeons, discussion topics presented in the course, and a sample of the end-of-course evaluation survey [file jamanetwopen-e2015675-s001.pdf]

Supplementary Online Content

Bryant TS, Carroll AL, Steinberg JR, et al. Implementation and evaluation of an educational program for increasing diversity and inclusion in surgery for preclinical students. *JAMA Netw Open*. 2020;3(9):e2015675. doi:10.1001/jamanetworkopen.2020.15675

eFigure. Course development timeline for "Service Through Surgery" including questions asked to surgeons, discussion topics presented in the course, and a sample of the end-of-course evaluation survey

This supplementary material has been provided by the authors to give readers additional information about their work.

© 2020 Bryant TS et al. *JAMA Network Open.*

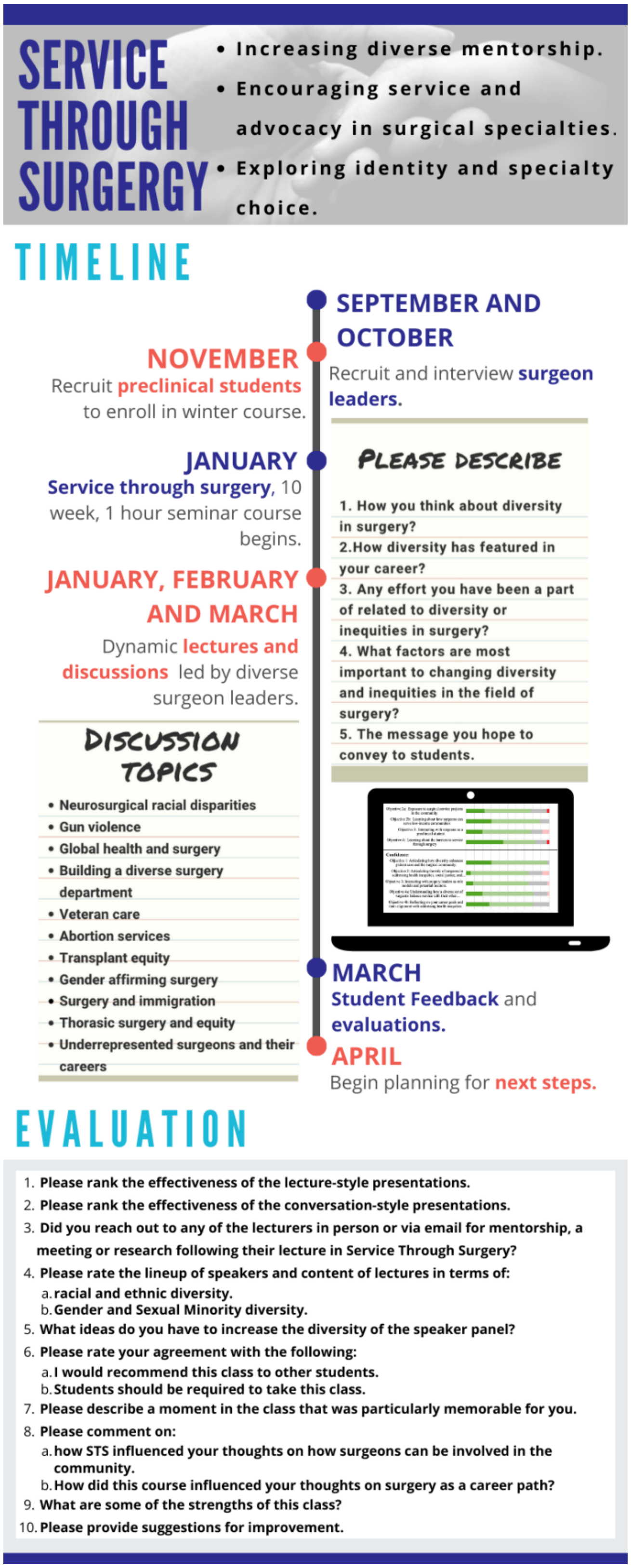

eFigure. Course development timeline for "Service Through Surgery" including questions asked to surgeons, discussion topics presented in the course, and a sample of the end-of-course evaluation survey.

© 2020 Bryant TS et al. *JAMA Network Open.*
